# Biosynthesized Chitosan-Coated Silver Nanoparticles: Insecticide Activity and Sublethal Effects Against *Drosophila suzukii* (Diptera: Drosophilidae)

**DOI:** 10.3390/biom15040490

**Published:** 2025-03-27

**Authors:** Daniel Martínez-Cisterna, Olga Rubilar, Lingyun Chen, Marcelo Lizama, Manuel Chacón-Fuentes, Andrés Quiroz, Pablo Parra, Ramón Rebolledo, Leonardo Bardehle

**Affiliations:** 1 Programa de Doctorado en Ciencias de Recursos Naturales, Facultad de Ciencias Químicas y Recursos Naturales, Universidad de La Frontera, Av. Francisco Salazar 01145, Casilla 54-D, Temuco 4811230, Chile; d.martinez11@ufromail.cl; 2 Centro de Investigación Biotecnológica Aplicada al Medio Ambiente (CIBAMA), Universidad de La Frontera, Av. Francisco Salazar 01145, Casilla 54-D, Temuco 4811230, Chile; 3 Laboratorio de Química Ecológica, Departamento de Ciencias Químicas y Recursos Naturales, Universidad de La Frontera, Av. Francisco Salazar 01145, Casilla 54-D, Temuco 4811230, Chile; andres.quiroz@ufrontera.cl; 4 Laboratorio de Entomología Aplicada, Facultad de Ciencias Agropecuarias y Medioambiente, Universidad de La Frontera, Av. Francisco Salazar 01145, Casilla 54-D, Temuco 4811230, Chile; m.lizama04@ufromail.cl (M.L.); p.parra03@ufromail.cl (P.P.); ramon.rebolledo@ufrontera.cl (R.R.); 5 Department of Chemical Engineering, Universidad de La Frontera, Av. Francisco Salazar 01145, Casilla 54-D, Temuco 4811230, Chile; 6 Department of Agriculture, Food and Nutritional Sciences, University of Alberta, Edmonton, AB T6G 2P5, Canada; lingyun1@ualberta.ca; 7 Programa de Doctorado en Ciencias Agroalimentarias y Medioambiente, Facultad de Ciencias Agropecuarias y Medioambiente, Universidad de La Frontera, Av. Francisco Salazar 01145, Casilla 54-D, Temuco 4811230, Chile; 8 Agriaquaculture Nutritional Genomic Center (CGNA), Temuco 4780000, Chile; manuel.chacon@cgna.cl; 9 Departamento de Producción Agropecuaria, Facultad de Ciencias Agropecuarias y Medioambiente, Universidad de La Frontera, Av. Francisco Salazar 01145, Casilla 54-D, Temuco 4811230, Chile

**Keywords:** green nanotechnology, fruit pest management, silver nanoparticles, chitosan coating, spotted-wing drosophila

## Abstract

The overuse of synthetic pesticides has triggered resistance in insect pests and caused severe environmental impacts, emphasizing the urgent need for sustainable alternatives in Integrated Pest Management (IPM). This study aimed to biosynthesize and characterize chitosan-coated silver nanoparticles (AgChNPs) using *Galega officinalis* leaf extract and evaluate their insecticidal effects against *Drosophila suzukii* (Diptera: Drosophilidae), a key pest of fruit crops worldwide. The biosynthesized AgChNPs (257.2 nm) were polydisperse, crystalline, and stable, as confirmed by UV-vis spectroscopy, dynamic light scattering (DLS), X-ray diffraction (XRD), and transmission electron microscopy (TEM). AgChNPs exhibited strong toxicity across multiple developmental stages. Combined larvicidal and pupicidal activity reached 48.3% and 73.3% at 500 and 1000 ppm, respectively, significantly affecting immature stages. As a consequence, adult emergence declined to 46.7%, 51.7%, and 26.7% at 250, 500, and 1000 ppm. Among emerged adults, 71.7% displayed sublethal effects, with 62.8% showing morphological malformations (deformed wings, dehydration) and 37.2% presenting cuticle demelanization. Adulticidal bioassays revealed progressive mortality over 48 h, with 96% mortality at 1000 ppm. Overall, AgChNPs caused acute and chronic toxicity, reduced adult emergence, and induced severe morphological alterations, demonstrating their potential as a sustainable nanotechnological tool for effective pest control within IPM programs.

## 1. Introduction

Agricultural areas affected by the discriminate pest attacks can experience significant reductions in crop yields, leading to substantial economic losses [1]. Among these pests, *Drosophila suzukii* (Diptera: Drosophilidae), commonly known as the spotted wing drosophila (SWD), is recognized as a major agricultural threat to small fruit crops worldwide [2]. The damage caused by SWD arises from the female’s ability to lay eggs in intact, ripening fruits using their serrated ovipositor. The larvae subsequently feed within the fruit, rendering it unmarketable [3]. The primary hosts of SWD include cherries, blueberries, blackberries, raspberries, and strawberries [4]. Globally, losses attributed to this insect range between 26% and 100% in cherry orchards. In Japan, economic losses of 80% to 100% have been recorded in cherries and grapes. In the United States, losses reported in 2008 amounted to $106 million and $405 million for cherries and berries, corresponding to 80% and 40% damage, respectively [5].

In Chile, SWD was first detected in 2017 through the capture of adult specimens in traps installed at the Mamuil Malal border crossing with Argentina (Villarrica-Pucón route, 39°34′43″ S, 71°29′16″ W) in the La Araucanía Region, southern Chile. Subsequently, the Agricultural and Livestock Service (SAG) confirmed its presence from the Tarapacá Region to the Aysén Region of the country [6,7]. Global and regional niche models indicate that 98.6% of SWD populations in Chile remain stable, while 1.54% are in the colonization phase. The climatic niche associated with stable populations encompasses 99% of suitable conditions, with minimal potential for expansion into novel climates (0.01%), suggesting a considerable risk of further invasion into Chilean territory [8]. For instance, field trials conducted by Buzzetti [9] in cherry orchards located in Cachapoal and Culenar, within the Ñuble Region, confirmed SWD infestations, with the first fruit losses reported in the country ranging from 10% to 15% of the total yield, resulting in economic losses between 5000 and 17,550 USD per hectare.

Currently, chemical control using synthetic pesticides remains the most widely employed strategy to combat SWD. However, its application is strictly regulated in many countries due to the associated environmental risks, including low nutrient use efficiency, soil degradation, water eutrophication, and rising economic costs [10,11]. As a result, nanotechnology has emerged as a promising alternative to reduce reliance on conventional pesticides and develop innovative strategies for Integrated Pest Management (IPM) [12], particularly through the application of biosynthesized chitosan-coated silver nanoparticles (AgChNPs).

In recent years, nanotechnology has attracted significant interest in agricultural due to its unique properties, including specialized surface structures, potent insecticidal effects, and environmental compatibility [13,14]. Biosynthesized AgChNPs are increasingly recognized as environmentally sustainable alternatives due to their ability to eliminate toxic reagents and minimize the use of harmful chemical compounds. AgNPs can be synthesized through biological systems, such as plant extracts, which are rich in bioactive compounds including flavonoids, alkaloids, terpenoids, and polyphenols, serving as natural reducing and stabilizing agents [15,16].

For instance, AgNPs have been biologically synthesized using *Galega officinalis*, a perennial herbaceous species from the Fabaceae family. This plant was selected for its high polyphenol and flavonoid content, demonstrating considerable potential for AgNP biosynthesis. This approach offers a cost-effective, scalable, and energy-efficient method for nanoparticle production, while significantly reducing environmental impact [17,18]. Additionally, chitosan (Ch) has been widely utilized due to its non-toxicity, biodegradability, and excellent bioavailability [19]. Chitosan contains a high density of free amino and hydroxyl groups, which interact with silver ions, enhancing nanoparticle stability, electro-optical properties, and biological activity [20].

AgNPs have been reported as effective agents for controlling various insect orders, including Lepidoptera, Coleoptera, Hemiptera, and Diptera [21]. Within Diptera, the order of primary interest in this study, AgNPs have demonstrated significant insecticidal effects. For instance, in *Musca domestica* (Diptera: Muscidae), AgNPs exhibited an LC_50_ of 3.64 mg/mL and LC_90_ of 7.74 mg/mL [22]. Additionally, AgNPs have been associated with physiological abnormalities in dipteran species, particularly *D. melanogaster*, including the induction of genotoxic activity, melanin loss, and developmental alterations [13,23,24,25]. Moreover, Ch has also been described as a potent natural insecticide, particularly against Diptera species of public health importance, such as *Culex quinquefasciatus*, *Aedes aegypti*, and *Anopheles stephensi* (Diptera: Culicidae), achieving LC_50_ values ranging from 12.27 to 14.62 µg/mL [14]. These findings suggest that AgNPs and Ch could serve as promising tool for controlling pest species within *Drosophilidae*. However, no studies have explored their potential against this insect family, including SWD.

Given the economic damage caused by SWD and the urgent need for environmentally friendly control alternatives, this study aims to biosynthesize AgChNPs and evaluate their insecticidal effects against SWD as an alternative to chemical pesticides.

## 2. Materials and Methods

### 2.1. Plant Collection

The leaves of *G. officinalis* were collected from rural areas surrounding the Maquehue sector in Temuco, La Araucanía Region, Chile. The collected leaves were thoroughly washed multiple times with potable water to remove dust and other impurities. Subsequently, they were air-dried under laboratory conditions at 25 °C for 24 h.

### 2.2. Biosynthesis of Silver Nanoparticles

Ten grams of *G. officinalis* leaves were placed in a 250 mL Erlenmeyer flask containing 100 mL of deionized water and boiled at 100 °C for 5 min. The resulting liquid extract was cooled to ambient temperature (25 ± 2 °C) and filtered using a vacuum pump with Whatman No. 1 filter paper.

Biosynthesized AgNPs was performed following the protocol described by Manosalva et al. [17]. The leaf extract was combined with 1.5 mM silver nitrate (AgNO_3_), and the solution’s pH was adjusted to 11. The reaction was maintained at room temperature (25 ± 2 °C) under continuous stirring using a magnetic stirrer for 24 h.

### 2.3. Biosynthesis of Chitosan-Coated Silver Nanoparticles

Biosynthesized AgNPs were treated with Ch (low molecular weight: 50,000–190,000 Da, with viscosity of 1% solution in 1% acetic acid ≤ 300 cP, and degree of acetylation of 75–85%, purchased from Sigma-Aldrich Co., St. Louis, MO, USA) following a modified version of the methodology proposed by Wulandari et al. [26]. AgNPs were mixed with 1% Ch solution in a 2:1 ratio, and the pH of the mixture was adjusted to 4. Ch was previously dissolved in 2% acetic acid. Once the AgChNPs were prepared, the solution was stirred continuously on a magnetic stirrer for 24 h to ensure homogenization.

### 2.4. Characterization of Biosynthesized AgChNPs

Bioreduction of biosynthesized AgChNPs was assessed using UV-visible absorption spectra across a range of wavelengths (200–800 nm) with a Genesys10s spectrophotometer (Thermo Scientific, Waltham, MA, USA), revealing a characteristic surface plasmon resonance (SPR) peak indicative of the successful formation of silver nanoparticles.

The average hydrodynamic diameter (nm), polydispersity index (PDI), and zeta potential were determined by dynamic light scattering (DLS) using a Zetasizer Nano Zs (Malvern Instruments Co, Malvern, UK). Measurements were conducted at 25 °C with a fixed angle of 173° in disposable folded capillary zeta cells with a 10 mm path length, using an aqueous suspension.

The crystalline structure of the biosynthesized AgChNPs was investigated using X-ray diffraction (XRD) analysis. Data were acquired with a Rigaku SmartLab X-ray diffractometer equipped with a Theta-Theta Bragg-Brentano goniometer and a solid-state D/teX Ultra 250 detector (Rigaku Corporation, Tokyo, Japan). XRD patterns were collected with Cu-Kα radiation (K = 1.5406 Å) at 30 kV and 40 mA, with Ni-filtered, in the 20–100° 2Theta range, counting 0.5°/s per step of 0.01°.

Morphology and size of AgChNPs were examined by Transmission Electron Microscopy (TEM) using a Talos F200C G2, Ceta 16M CMOS Camara (Thermo Fisher Scientific, Hillsboro, OR, USA); pixel size 14 μm × 14 μm, 16 bit.

### 2.5. Rearing of SWD

Specimens of SWD were obtained from an established colony maintained at the Entomology Laboratory of Biofuturo Ltd.a, Fundo Alianza Km 11, Traiguen-Galvarino, Region de La Araucanía, Chile. The specimens were reared under controlled laboratory conditions at the Laboratory of Ecological Chemistry, Universidad de La Frontera, Temuco, Chile. Environmental conditions were maintained at 22 °C, 40% relative humidity, and a 12:12 light:dark photoperiod, following the protocol described by Kalajdzic & Schetelig [27].

The flies were fed an artificial diet adapted from Dalton [28]. The diet consisted of agar (8 g), brewer’s yeast (40 g), wheat germ (80 g), granulated sugar (100 g), and distilled water (1 L). The mixture was heated to boiling under continuous stirring and then allowed to cool to 60 °C. Once cooled, propionic acid (3 mL), ethanol (8 mL), and methylparaben (0.8 g) were added to the mixture. Subsequently, they were stored in a refrigerator at 4 °C until use.

### 2.6. Insecticidal Effect of Biosynthesized AgChNPs

#### 2.6.1. Chronic Toxicity

##### Larvicidal Bioassay

The larvicidal activity of biosynthesized AgChNPs was evaluated using an artificial diet prepared in plastic cups (50 mL). The diet before mentioned was supplemented with different concentrations of biosynthesized AgChNPs (T1: 100 ppm, T2: 250 ppm, T3: 500 ppm y T4: 1000 ppm). Subsequently, three 2nd instar larvae of SWD were introduced into each plastic cup. Each concentration was tested in 20 replicates, with three larvae per replicate (total *n* = 360). Deionized water (H_2_O_d_) was used as negative control (C^−^) and AgNO_3_ as positive control (C^+^).

Larval mortality was assessed every 48 h over a 26-day period. The percentage mortality was calculated using Abbott’s formula [29]:

$$\mathrm{Percentage of mortality}=\frac{\mathrm{Number of dead individuals}}{\mathrm{Number of dead individuals}}\times 100$$

The bioassay was conducted under controlled conditions at 24 °C, 40% relative humidity, and a 12:12 light-to-dark photoperiod.

##### Pupicidal Activity

The surviving larvae were continuously fed with the treated diet from the larvicidal bioassay until they developed into pupae and subsequently emerged as adults. Pupicidal activity was determined by calculating the difference between the total number of pupae and the number of successfully emerged adults.

#### 2.6.2. Acute Toxicity

##### Adulticidal Bioassay

The adulticidal activity of biosynthesized AgChNPs was evaluated using recently emerged SWD adults obtained from the laboratory-reared colony. Five flies were placed in a Petri dish (100 mm) and sprayed with 500 µL of biosynthesized AgChNPs. Each concentration was tested in 15 replicates, resulting in a total sample of 450 individuals. Mortality was evaluated every 24 h for 2 days.

### 2.7. Statistical Analysis

The statistical software IBM SPSS Statistics 20 (IBM Corp., Armonk, NY, USA) was used to analyze the larvicidal and pupicidal activity, the number of hatched flies, and the adulticidal activity. A one-way analysis of variance (ANOVA) followed by Duncan’s multiple range test was performed to determine significant differences between treatment groups (*p* ≤ 0.05). Data are presented as mean ± SE, with different letters above bars indicating significant differences. Additionally, differences in the proportions of sublethal effects, such as malformations and demelanization, were assessed using a Chi-squared test, with significance set at *p* ≤ 0.05.

## 3. Results and Discussion

### 3.1. Characterization of AgChNPs

#### 3.1.1. UV-Vis of AgChNPs

**Figure 1:**
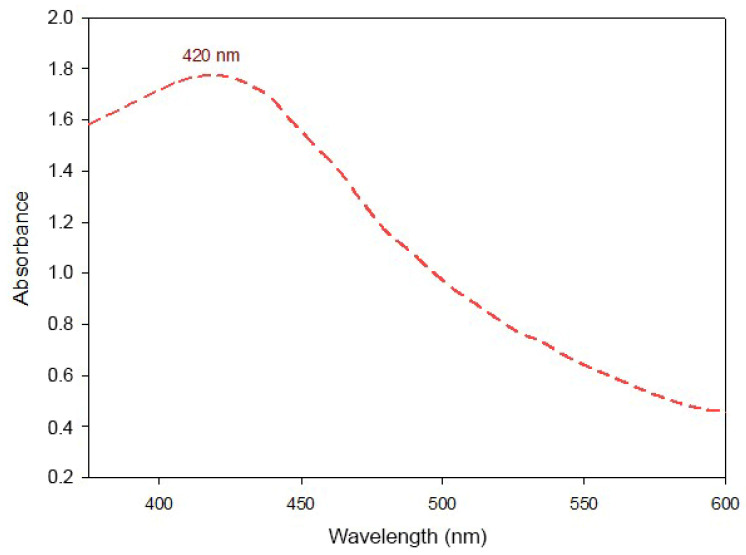
UV-vis absorption spectrum of biosynthesized AgChNPs, highlighting the characteristic SPR peak.

The surface plasmon resonance (SPR) of AgChNPs was characterized using UV-vis spectroscopy, which revealed a maximum absorption peak at 420 nm (Figure 1). This peak corresponds to the excitation of characteristic surface plasmon resonance vibrations of AgNPs, confirming the successful biosynthesis of AgChNP nanocomposites [30]. These findings align with those reported by Cortés et al. [31], who also reported SPR peaks at 420 nm in AgChNPs biosynthesized from *Aloe barbadensis* (Asparagales: Asphodelaceae), further validating the synthesis process. Additionally, Wulandari et al. [26] reported that SPR peaks within the 410–450 nm range are typically associated with nanoparticles of spherical morphology, as demonstrated in their study on AgChNPs biosynthesized from *Musa paradisiaca* (Zingiberales: Musaceae).

#### 3.1.2. Hydrodynamic Diameter (nm), PDI, and Z Potential

**Figure 2:**
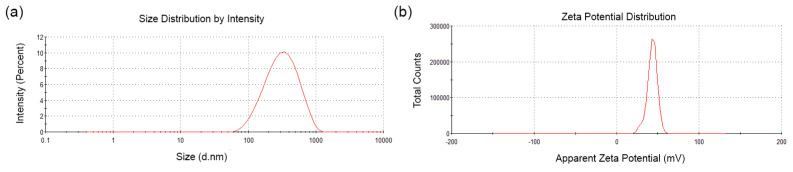
Size and zeta potential of AgChNPs. (**a**) Average size distribution by intensity. (**b**) Zeta potential distribution of AgChNPs.

The average hydrodynamic diameter of the AgChNPs was 257.2 nm (Figure 2a), with a polydispersity index (PDI) of 0.26 and a zeta potential of +43.3 mV (Figure 2b). A PDI value of 0.26 suggests a relatively narrow size distribution in solution. However, this does not necessarily indicate monodispersity at the individual particle level, as confirmed by TEM analysis. Zeta potential values less than −30 mV and greater than +30 mV are considered ideal for the particles to prevent aggregation [32]. These results are consistent with the findings by Karuppaiah et al. [33], who reported an increase in AgNP nanoparticle size following Ch coating, with particles measuring 545 nm, a PDI of 0.26, and a zeta potential of +37.5 mV after Ch incorporation. These observations indicate that the Ch shell introduces a positive surface charge, resulting in a positive zeta potential.

#### 3.1.3. X-Ray Diffraction (XRD) of AgChNPs

**Figure 3:**
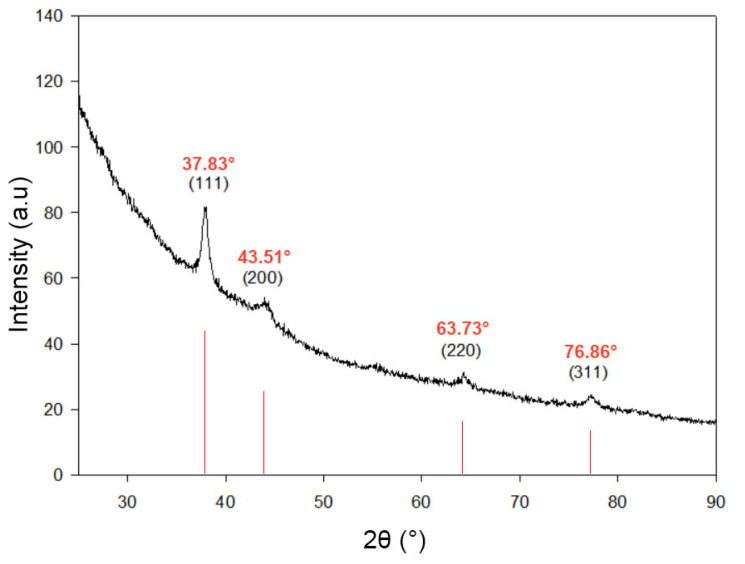
XRD pattern of biosynthesized AgChNPs.

The crystalline nature of the nanoparticles was determined through XRD analysis. The XRD patterns exhibited intense peaks corresponding to Bragg reflections at 2θ = 37.83, 43.51, 63.73, and 76.86 (Figure 3), which are associated with the crystal facets of (111), (200), (220), (311) of the face-centered cubic structure of metallic Ag. The sharp appearance of these peaks confirmed the crystalline structure of the AgNPs, while the broad nature of the XRD peaks indicated the nanoscale size of the particles [34,35].

#### 3.1.4. TEM Analysis of AgChNPs

**Figure 4:**
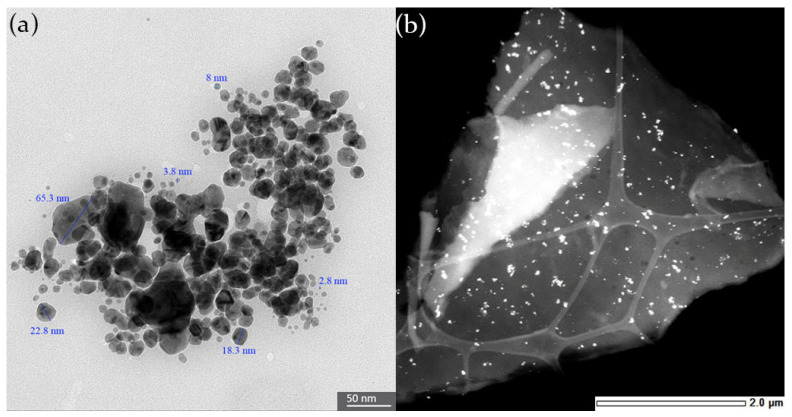
TEM pictures. The image revealed sizes ranging from 2.8–65.3 nm showing the morphological characteristics of (**a**) biosynthesized AgNPs and, (**b**) biosynthesized AgChNPs.

TEM analysis confirmed that both AgNPs and AgChNPs exhibited polydispersity, with spherical structures ranging in size from 2.8 to 65.3 nm (Figure 4a,b). This contrasts with the DLS results, which indicate a hydrodynamic diameter of 257.2 nm in solution, highlighting the tendency of nanoparticles to aggregate or form larger complexes in solution. Similar characteristics were reported by Seekonda and Rani [36], who observed AgNPs with an irregular spherical shape of varying sizes biosynthesized using *Embelia robusta* (Ericales: Primulaceae) extract. These findings are consistent with other studies that obtained AgNPs via biosynthesis using aqueous extracts from various terrestrial plants [37,38]. For instance, AgNPs produced from *Baugainvillea glabra* (Caryophyllales: Nyctaginaceae) exhibited a size range of 8–90 nm [39].

Additionally, structures with varying contrasts were observed surrounding the AgNPs following chitosan coating (Figure 4b). These variations are likely attributable to the presence of organic residues derived from the leaf extract, which may act as organic capping agents. These agents incorporate hydroxyl (-OH) and amino (-NH_2_) groups from chitosan, as previously described by De Matteis et al. [40], who demonstrated that such residues stabilize nanoparticles by forming a capping layer through surface interactions, thereby enhancing stability and biocompatibility.

### 3.2. Chronic Toxicity

#### 3.2.1. Larvicidal Bioassay

Biosynthesized AgChNPs exhibited significant larvicidal activity against 2nd-instar larvae of SWD (Table 1). The highest concentration (T4) resulted in a larval mortality rate of 43.3%, which was significantly higher than that observed in the other treatments and the negative control group (*p* = 0.001). In contrast, the positive control achieved 100% larval mortality. Lower concentrations (T1, T2, and T3) displayed minimal larvicidal activity, with mortality rates of 10%, 3.3%, and 3.3%, respectively, showing no significant differences compared to the negative control.

These findings align with previous studies on the insecticidal effects of AgNPs against agricultural pests. For instance, strong toxic effects of AgNPs have been reported for *Plutella xylostella* (Lepidoptera: Plutellidae) (LC_50_ 0.691 mg/mL) [41], *Spodopttera litura* (Lepidoptera: Noctuidae) (LC_50_ 0.0312 mg/L–46.9 mg/L), *Earias vittela* (Lepidoptera: Noctuidae) (LC_50_ 25.12 mg/L–45.46 mg/L), *Bombyx mori* (Lepidoptera: Bombycidae) (100% mortality at 1600–3200 mg/L), *Agrotis ipsilon* (Lepidoptera: Noctuidae), and *Trichoplusia ni* (Lepidoptera: Noctuidae) (LC_50_ 5.20 mg/mL and LC_50_ 0.81 mg/mL respectively), with toxic effects primarily observed in larval stages [42,43,44,45,46].

Additionally, a repellency effect was observed during the larvicidal bioassay (Table 1). After 48 h of treatment, 81.6% of the larvae at the highest concentration (T4) moved outside the diet, indicating a pronounced repellency effect. This behavior was significantly different from that observed at the lower concentration (T1, 8.3%) and in the negative control group (3.3%) (*p* = 0.001). These findings suggest that the observed repellency constitutes a distinct behavioral response from mortality, potentially providing additional insights into the mode of action of AgChNPs and warranting further investigation.

Similar findings were reported by Rehman et al. [47], who observed a dose-dependent repellency effect of green-synthesized AgNPs against *Oryzaephilus surinamensis* (Coleoptera: Silvanidae) and *Sitophilus granarius* (*Coleoptera: Curculionidae*). The highest repellency (42.7%) was recorded in *O. surinamensis* at 500 ppm, while *S. granarius* exhibited lower repellency (36.2%) under the same conditions. These results support our observations, suggesting that nanoparticle concentration and target species influence repellency. Similarly, Manimegalai et al. [48] reported antifeedant activity of 78.77% and 82.16% against *S. litura* and *Helicoverpa armigera* (Lepidoptera: Noctuidae), respectively, with maximum larval mortality rates of 78.49% and 72.70% at 150 mg/L.

Interestingly, the concentrations used in this study were generally lower than those reported for Lepidopteran pests, yet they exhibited notable larvicidal activity. This enhanced efficacy could be attributed to the chitosan coating, which is known to improve nanoparticle bioavailability, thereby increasing their insecticidal potential. Supporting this, chitosan-silver nanoparticles have demonstrated significant mosquitocidal activity against other Diptera species, such as *A. stephensi*, *A. aegypti*, and *C. quinquefasciatus*, with LC_50_ values of 10.24, 11.35, and 12.43 ppm, respectively [49]. These nanoparticles exhibited a remarkable ability to cross biological barriers and induce adverse physiological effects [50], underscoring the potential of biosynthesized AgChNPs as highly effective larvicidal agents.

#### 3.2.2. Pupicidal Activity

**Figure 5:**
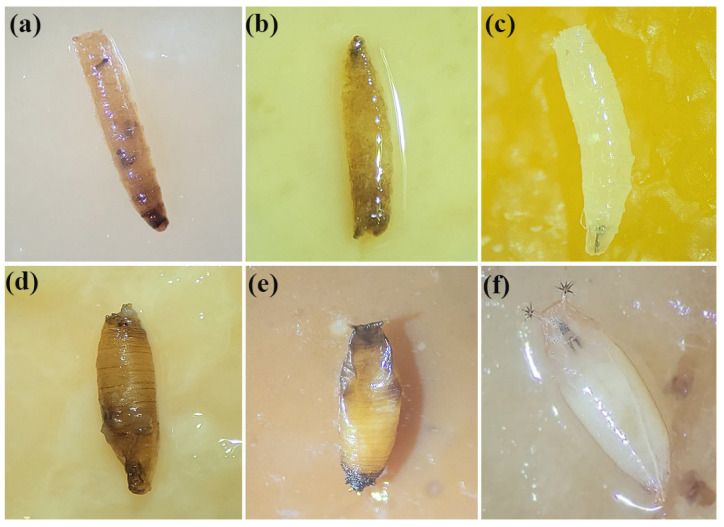
*Drosophila suzukii* individuals subjected to treatment with biosynthesized AgChNPs at 1000 ppm. (**a**,**b**) 2nd-instar larvae after treatment with biosynthesized AgChNPs. (**c**) Untreated healthy larvae. (**d**,**e**) Pupae after treatment with biosynthesized AgChNPs. (**f**) Untreated healthy pupae.

The biosynthesized AgChNPs exhibited significant pupicidal activity against SWD individuals that pupated following exposure in the larvicidal bioassay (Table 1). The highest pupicidal activity was observed at concentrations of 250 ppm (T2, 48.3%), and 500 ppm (T3, 45%), showing significant differences compared to the negative control and other treatments (*p* = 0.001). At these concentrations, larvicidal activity was minimal (3.3%), suggesting that larvae surviving exposure to biosynthesized AgChNPs, may experience developmental disruptions, including mortality during the pupal stage, deformed teguments, and dark pigmentation (Figure 5). The lack of high pupal mortality at the highest concentration (1000 ppm) could be attributed to increased larval mortality, resulting in fewer individuals reaching the pupal stage. However, total mortality (including both larvicidal and pupicidal activity) reached 48.3% and 73.3% at 500 and 1000 ppm, respectively, highlighting the strong impact on immature stages.

Similar findings have been reported using biosynthesized AgNPs against *D. melanogaster* and *Musca domestica*, with concentrations ranging from 10 to 200 ppm causing multiple effects, including disruption of pupal development, reductions in larval hatching rates (50% and 80% at 20 ppm and 40 ppm, respectively), and high mortality rates (97%) [23,25,51]. Additionally, Manimegalai et al. [48] observed morphological abnormalities in *S. litura* and *H. armigera*, including alterations in epithelial layer alignment and significant changes in the brush border and gut lumen. These anomalies are believed to result from the impact of AgNPs on copper transporter channels, which interfere with insect development [52].

#### 3.2.3. Adult Flies Emerged

The total number of emerged flies across all treatments was 120, corresponding to 37.5% of the total individuals exposed to AgChNPs. Emergence rates were subsequently evaluated (Table 2), revealing significant differences between the 1000 ppm treatment (T4) and the negative control group, with emergence rates of 26.7% and 76.7%, respectively (*p* = 0.001). The 100 ppm treatment (T1) showed an emergence rate of 81.7%, with no significant differences compared to the negative control. In contrast, the 250 ppm (T2) and 500 ppm (T3) treatments resulted in emergence rates of 46.7% and 51.7%, respectively. Notably, the 1000 ppm (T4) concentration led to a marked reduction in adult emergence.

**Figure 6:**
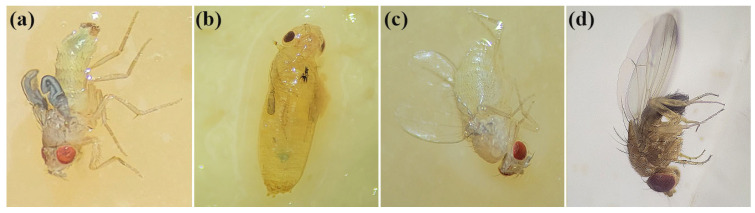
*Drosophila suzukii* flies that completed their life cycle after being treated with biosynthesized AgChNPs. (**a**,**b**) Adult malformed after treatment with biosynthesized AgChNPs (1000 ppm). (**c**) Adult with demelanization after treatment with biosynthesized AgChNPs (1000 ppm). (**d**) Untreated healthy flies.

Furthermore, 71.7% of the emerged flies exhibited significant sublethal effects. Among these, malformations were observed in 62.8% of individuals, while demelanization occurred in 37.2% of affected flies (Table 2). Statistical analyses revealed significant differences between the proportions of malformations and demelanization (Chi-squared test: χ^2^ = 7.99, *p* = 0.0047). Sublethal effects primarily included morphological abnormalities such as exoskeletal deformities, malformed wings, incomplete emergence from the pupal stage, a dehydrated appearance, and white cuticle coloration (Figure 6).

Similar effects have been reported by Panacek et al. [23] in *D. melanogaster*, where exposure to AgNPs at 10 ppm resulted in a slight reduction in adult pigmentation. At 20 ppm, a 50% decrease in the number of hatched individuals was observed, accompanied by significant reductions in body pigmentation. Additionally, long-term exposure to AgNPs affected *Drosophila* fertility over the first three filial generations. These effects have been associated with nanoparticle toxicity within the organism, which disrupts key enzymes involved in catabolic processes, including dopamine (DA), octopamine (OA), juvenile hormone (JH), and ecdysteroids such as 20-hydroxyecdisone (20HE) [23,53].

### 3.3. Acute Toxicity

#### Adulticidal Bioassay

**Figure 7:**
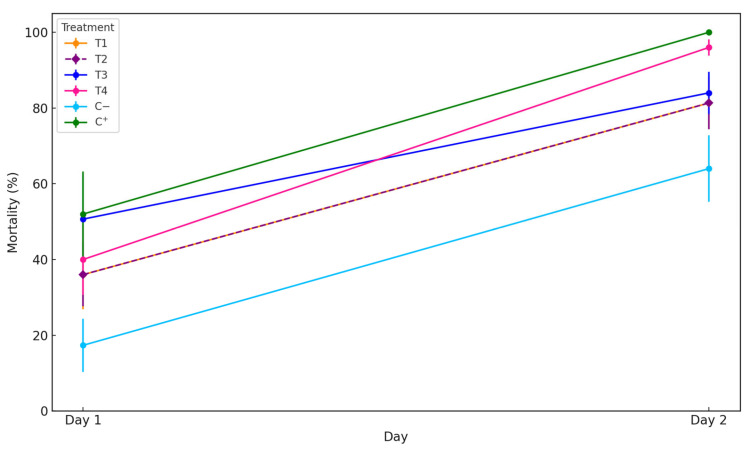
Adulticidal bioassay. Mortality percentage of adult individuals exposed to AgChNPs treatments. T1: 100 ppm, T2: 250 ppm, T3: 500 ppm, T4: 1000 ppm, C^−^: H_2_O_d_, C^+^: AgNO_3_. Different letters indicate significant differences (*p* < 0.05) based on ANOVA test following Duncan’s test.

Biosynthesized AgChNPs exhibited strong insecticidal activity against adult SWD flies after 48 h of exposure (Figure 7). All tested concentrations resulted in high mortality rates: 81.3%, 81.3%, 84%, and 96% for the concentrations of 100, 250, 500, and 1000 ppm, respectively. The highest concentration (1000 ppm) achieved mortality levels comparable to the positive control (+), which exhibited 100% mortality. All treatments showed significant differences compared to the negative control (−), which presented a mortality rate of 64% (*p* = 0.006), a relatively high value that may be attributed to environmental factors, handling stress, or natural mortality.

The observed insecticidal activity of AgChNPs against DSW is likely mediated by multiple mechanisms, including oxidative stress induction, disruption of cellular membranes, and interference with key physiological pathways, as previously reported for silver-based nanomaterials [21]. The high mortality rates across all concentrations suggest a potent mode of action, potentially enhanced by the chitosan coating, which may improve nanoparticle stability, bioavailability, and interaction with insect cuticles [54].

When compared to other studies on dipteran pests, AgChNPs have demonstrated strong efficacy, with LC_50_ values of 9.671 ppm for *A. stephensi*, 12.015 ppm for *A. aegypti*, and 12.965 ppm for *C. quinquefasciatus* [49]. The higher concentrations required for DSW may be explained by species-specific differences in cuticular structure, enzymatic detoxification pathways, or behavioral factors that reduce direct contact with the nanoparticles. Overall, these findings support the potential of AgChNPs as effective agents for dipteran pest control.

## 4. Conclusions

In this study, AgChNPs were successfully biosynthesized and characterized. DLS analysis indicated that AgChNPs exhibited a relatively narrow size distribution in solution and were stable, as supported by a zeta potential. However, TEM analysis revealed significant polydispersity at the individual particle level. UV-vis spectroscopy confirmed the characteristic SPR spectrum of silver, while XRD analysis identified Bragg’s reflections corresponding to the face-centered cubic structure of AgChNPs, and TEM analysis revealed their spherical morphology.

Regarding the toxicological effects of AgChNPs on SWD, the biosynthesized AgChNPs exhibited both acute and chronic insecticidal activity. They demonstrated high toxicity against larvae and adults, significantly reducing adult emergence rates compared to the negative control. In addition to inducing mortality, AgChNPs caused developmental abnormalities, including malformations and demelanization, underscoring their potential to disrupt the pest’s lifecycle.

These findings underscore the potential of AgChNPs as an environmentally sustainable alternative for pest control. Their integration into Integrated Pest Management (IPM) strategies could enhance pest management efficiency by reducing reliance on conventional pesticides and promoting sustainable agricultural practices. However, further research is needed to elucidate the specific mechanisms of action of AgChNPs, including their effects on target pest physiology, gene expression, and hormonal regulation.

## Figures and Tables

**Table 1:** Percentage mortality of larvicidal and pupicidal activities, and total mortality treatments of AgChNPs on DSW.

Test Sample	Concentration (ppm)	Repellency (%)	Larvicidal Activity (%)	Pupicidal Activity (%)	Total Mortality (%)
AgChNPs	100	8.3 ± 3.1 ^a^	10 ± 4.9 ^a^	8.3 ± 3.3 ^a^	18.3 ± 6.6 ^a^
	250	43.3 ± 8.8 ^c^	3.3 ± 3.3 ^a^	48.3 ± 7.0 ^d^	51.7 ± 7.4 ^b^
	500	24.9 ± 5.9 ^b^	3.3 ± 2.3 ^a^	44.9 ± 7.4 ^cd^	48.3 ± 7.4 ^b^
	1000	81.6 ± 7.4 ^d^	43.3 ± 4.9 ^b^	29.9 ± 3.3 ^bc^	73.3 ± 3.9 ^c^
Control +	AgNO_3_	0 ± 0	80 ± 0 ^c^	20 ± 7.0 ^ab^	100 ± 0 ^d^
Control −	H_2_O_d_	3.3 ± 2.3 ^a^	3.3 ± 2.3 ^a^	19.9 ± 5.6 ^ab^	23.3 ± 5.5 ^a^

Within each column, means ± SE followed by different letters indicate significant differences (ANOVA, Duncan HSD test, *p* ≥ 0.05).

**Table 2:** Mean percentage of hatched flies (%) and sublethal effects after exposure AgChNPs treatments.

Test Sample	Concentration (ppm)	Hatched Flies (%)	Malformations (%)	Demelanization (%)
AgChNPs	100	81.7 ± 6.6 ^d^	30 ± 8.7 ^b^	18.3 ± 6.6 ^b^
	250	46.7 ± 7.8 ^c^	19.9 ± 7.4 ^b^	10 ± 4.8 ^ab^
	500	51.7 ± 7.4 c	23.3 ± 6.4 ^b^	6.6 ± 3.0 ^ab^
	1000	26.6 ± 3.9 ^b^	16.6 ± 5.1 ^ab^	14.9 ± 5.1 ^b^
Control +	AgNO_3_	0 ± 0 ^a^	0 ± 0 ^a^	0 ± 0 ^a^
Control −	H_2_O_d_	76.7 ± 5.5 ^d^	0 ± 0 ^a^	0 ± 0 ^a^
Total affected flies	All concentrations	71.7	62.8 ^a^	37.2 ^b^

Within each column, means ± SE followed by different letters indicate significant differences (ANOVA, Duncan HSD test, *p* ≥ 0.05). Sublethal effects were analyzed using a Chi-squared test (χ^2^ = 7.99, *p* = 0.0047).

## Data Availability

Data are contained within the article.
